# Morphologic Alterations Precede Functional Hepatic Impairment as Determined by ^13^C-Methacetin Liver Function Breath Test in Adult Fontan Patients

**DOI:** 10.3389/fcvm.2021.764009

**Published:** 2021-12-23

**Authors:** Anastasia Schleiger, Peter Kramer, Hannes Sallmon, Niklas Jentsch, Marta Pileckaite, Friederike Danne, Marie Schafstedde, Hans-Peter Müller, Tobias Müller, Frank Tacke, Maximilian Jara, Martin Stockmann, Felix Berger, Stanislav Ovroutski

**Affiliations:** ^1^Department of Congenital Heart Disease/Pediatric Cardiology, Deutsches Herzzentrum Berlin, Berlin, Germany; ^2^Institute for Cardiovascular Computer-Assisted Medicine, Charité—Universitätsmedizin Berlin, Berlin, Germany; ^3^Berlin Institute of Health, Berlin, Germany; ^4^Charité Centre for Internal Medicine and Dermatology, Berlin, Germany; ^5^Department of Gastroenterology and Hepatology, Charité—Universitätsmedizin Berlin, Berlin, Germany; ^6^Department of General, Visceral and Vascular Surgery, Charité—Universitätsmedizin Berlin, Berlin, Germany; ^7^Department of Pediatric Cardiology, Charité—Universitätsmedizin Berlin, Berlin, Germany; ^8^German Centre for Cardiovascular Research (DZHK), Berlin, Germany

**Keywords:** Fontan-associated liver disease, enzymatic liver function, hepatic assessment, second-organ dysfunction, Fontan failure

## Abstract

**Objectives:** Fontan-associated liver disease (FALD) is the most common end-organ dysfunction affecting up to 70–80% of the Fontan population. The clinical significance of FALD is incompletely understood and no unambiguous correlation between hepatic function and FALD severity has been established. In this study, we sought to evaluate maximal liver function capacity with liver maximum function capacity test (LiMAx®) in adult Fontan patients.

**Methods:** Thirty-nine adult Fontan patients (median age: 29.4 years [IQR 23.4; 37.4], median follow-up after Fontan operation: 23.9 years [IQR 17.8;26.4]) were analyzed in a cross-sectional observational study using LiMAx® test (Humedics GmbH, Berlin, Germany), laboratory testing, transient elastography (TE) and hepatic ultrasound. The LiMAx® test is based on the metabolism of ^13^C-methacetin, which is administered intravenously and cleaved by the hepatic cytochrome P4501A2 to paracetamol and ^13^CO_2_, which is measured in exhaled air and correlates with maximal liver function capacity.

**Results:** Maximal liver function capacity assessed by LiMAx® test was normal in 28 patients (>315 μg/h^*^kg) and mildly to moderately impaired in 11 patients (140–314 μg/h^*^kg), while no patient displayed severe hepatic impairment (<139 μg/kg^*^h). No correlation was found between maximal liver function capacity and hepatic stiffness by TE (*r*^2^ = −0.151; *p* = 0.388) or the presence of sonographic abnormalities associated with FALD (*r*^2^ = −0.204, *p* = 0.24). There was, however, an association between maximal liver function capacity and the laboratory parameters bilirubin (*r*^2^ = −0.333, *p* = 0.009) and γ-glutamyl transferase (*r*^2^ = −0.367; *p* = 0.021). No correlation was detected between maximal liver function capacity and the severity of FALD (*r*^2^ = −0.235; *p* = 0.152).

**Conclusion:** To the best of our knowledge, this is the first study to evaluate maximal liver function capacity using LiMAx® test in Fontan patients, which is a useful complementary diagnostic instrument to assess chronic hepatic injury. Maximal liver function capacity was preserved in most of our adult Fontan patients despite morphologic evidence of FALD. Moreover, maximal liver function capacity does not correlate with the extent of FALD severity evaluated by sonography or laboratory analysis. Thus, the development and progression of FALD in Fontan patients is not a uniform process and diagnostics of chronic hepatic injury during follow-up should encompass various modalities.

## Introduction

Over the past decades, survival of Fontan-palliated patients significantly improved with the majority of patients now reaching adulthood (1, 2). Nevertheless, the unphysiological Fontan circulation leads to progressive end-organ damage in the long-term (3–5). Fontan-associated liver disease (FALD) is the most common end-organ dysfunction and affects up to 70–80% of the adult Fontan population (5, 6). FALD manifestations vary from slightly elevated serum liver enzymes and mild hepatic parenchymal changes to end-stage liver cirrhosis (5, 6). The clinical significance of FALD is incompletely understood, and its diverse manifestations need to be put into clinical context when making therapeutic decisions. In a previous study, we proposed a scoring system (FALD score) to grade FALD severity based on a combination of laboratory parameters, hepatic ultrasound and transient elastography (TE). Our results revealed that the FALD score significantly correlated with Fontan hemodynamics and reliably discriminated between patients with and without Fontan failure (7). Liver-associated morbidity and mortality are well-described in the adult Fontan population and constitute major risk factors limiting survival after cardiac transplantation (8, 9). Therefore, reliable diagnostic modalities are indispensable to monitor hepatic end-organ damage and to determine the optimal timing for cardiac transplantation.

Since conventional liver function tests are often insensitive indicators of early disease stages, Stockmann et al. introduced the liver maximum capacity test (LiMAx®) for exact determination of quantitative enzymatic liver function and prediction of outcome after hepatectomy (10). The test is based on the enzymatic function of the cytochrome P4501A2 (CYP1A2) system, which is exclusively expressed in hepatocytes and is proportional to the hepatic parenchymal volume (10). As methacetin is exclusively metabolized by CYP1A2, the intravenous administration of ^13^C-methacetin and the continuous real-time breath analysis of exhaled ^13^CO_2_ provides an exact quantification of maximal liver capacity.

The aims of this study were to determine maximal liver function capacity with the LiMAx® test in adult Fontan patients, in comparison to the results of other hepatic diagnostic modalities and to analyze its relationship with the severity of FALD.

## Methods

### Study Design and Patients

From 2019 to 2021 we performed a cross-sectional observational study including 39 adult Fontan patients, who successively presented in our outpatient clinic for follow-up and received measurement of maximal liver function capacity using the LiMAx® test. Exclusion criteria consisted of patient age <18 years and/or intolerance to paracetamol or methacetin. The study was approved by the institutional review board and ethics committee (decision number: EA2/127/18). Informed written consent was obtained from all individual participants prior to inclusion.

### FALD Diagnostics

Our institutional protocol and diagnostic algorithm for hepatic assessment of Fontan palliated patients has previously been described in detail and consists of laboratory analyses, hepatic ultrasound and liver stiffness measurement by TE (5). The laboratory parameters alanine-aminotransferase (ALT), γ-glutamyltransferase (γGT), total bilirubin, α_2_-macroglobulin, apolipoprotein A_1_ and haptoglobin were required to calculate a biomarker fibrosis score with FibroTest®. FibroTest® was computed on Biopredictive website (Paris, France; www.biopredictive.com). The calculated Fibrotest® score was converted into liver fibrosis stages according to METAVIR histological classification for liver biopsies (11). The calculation of the FALD score has previously been described in detail (7). Briefly, scoring points assigned for each hepatic abnormality detected in the diagnostics mentioned above were summed up for the final FALD score (7). In addition, patients received a standardized LiMAx® test as described by Stockmann et al. (10). Briefly, after 4 h of fasting, patients were placed in a resting horizontal position. Ten minutes prior to the injection of ^13^C-methacetin, the baseline ^13^CO_2_/^12^CO_2_ ratio was recorded. A solution of 2 mg/kg body weight ^13^C-labeled methacetin was intravenously injected as a bolus over a maximum of 30 s followed by 20 mL 0.9% sodium chloride solution. ^13^C-methacetin is metabolized by the hepatozyte-specific CYP1A2 system into paracetamol and ^13^CO_2_, which is exhaled and measured in expired air (Supplementary Figure 1). Each LiMAx® test analyzed 46 breath samples per patient and the result is given in μg/kg^*^h (Supplementary Figure 2).

## Statistical Analysis

Data were obtained from medical records of the German Heart Centre Berlin. Patients' characteristics were expressed as median and interquartile range [IQR]. Fontan follow-up duration was defined as the interval between Fontan operation and last follow-up. Correlations between maximum liver capacity and laboratory parameters, TE, the number of hepatic abnormalities detected by ultrasound and the FALD score were assessed using Spearman's correlation. Associations between FALD severity graded as mild, moderate, and severe and maximal liver function capacity was analyzed using Kuskal Wallis and Wilcoxon tests as appropriate. Statistical analyses were performed using SPSS statistical software (version 23, IBM Corp., NY, USA). A *p* < 0.05 was considered statistically significant.

## Results

Patient characteristics of the entire cohort are listed in Table 1. Median patient age was 29.4 years [IQR 23.4, 37.4] and median follow-up after Fontan operation 23.9 years [IQR 17.8, 26.4]. The most common underlying cardiac morphologies were tricuspid atresia (*n* = 10), double inlet left ventricle (*n* = 11) and unbalanced atrioventricular septal defect (*n* = 3). Fontan modifications included extracardiac conduit in 13 patients, lateral tunnel in 16 patients and atriopulmonary/ atrioventricular connection (APC/AVC) or other modifications in 10 patients. Systolic ventricular function was normal or mildly impaired in the majority of the cohort (*n* = 33). Atrioventricular valve regurgitation was classified as absent or mild in 30 patients, moderate in 8 patients and severe in 1 patient. In 4 patients cardiac transplantation was performed, 2 patients died after transplantation. In addition, 1 patient died on mechanic circulatory support prior to cardiac transplantation.

**Table 1:** Patient characteristics.

Patient age (years)	29.4 [23.4;37.4]
Age at Fontan operation (years)	6.5 [3.5;12.9]
Follow-up after Fontan (years)	23.9 [17.8;26.4]
**Cardiac anatomy**	
Double inlet left ventricle	11 (28.2%)
Tricuspid atresia	10 (25.6%)
Unbalanced AVSD	3 (25.6%)
Complex TGA	3 (25.6%)
Hypoplastic left heart syndrome	2 (5.1%)
Other	10 (15.5%)
Left ventricular morphology	27 (69.2%)
**Fontan type**	
Intracardiac TCPC	16 (41.7%)
Extracardiac TCPC	13 (33.3%)
APC/AVC/other	10 (30.3%)
**Impairment of systolic ventricular function**	
None	12 (30.8%)
Mild	21 (53.8%)
Moderate	5 (12.8%)
Severe	1 (2.6%)
**Atrioventricular valve insufficiency**	
None	13 (33.3%)
Mild	17 (43.6%)
Moderate	8 (20.5%)
Severe	1 (2.6%)

*Data are presented as median [IQR] or frequencies (%)*.

*APC, Atriopulmonary connection; AVC, Atrioventricular connection; AVSD, Atrioventricular septal defect; IQR, interquartile range; TCPC, total cavopulmonary connection; TGA, Transposition of great arteries*.

### Hepatic Assessment

Results from laboratory analysis, TE, hepatic ultrasound and the LiMAx® test are reported in Table 2. According to age-adjusted institutional reference values, ALT was elevated in 6 patients, aspartate aminotransferase (AST) in 7 patients, γGT in 31 patients and bilirubin in 13 patients. Thrombocytopenia was found in 12 patients. Median Fibrotest® fibrosis score was 0.6 [IQR 0.4, 0.6]. Referring to Fibrotest® calculation, fibrosis was staged F2 in the majority of our patients (*n* = 12; Table 2). Ultrasound revealed hepatic parenchymal changes in all patients (Table 2). The most common ultrasound findings were heterogeneous echotexture (*n* = 35), liver vein dilatation (*n* = 31), altered liver vein morphology (*n* = 25) and segmental hypertrophy or atrophy (*n* = 11). Hyperechogenic lesions were present in 7 patients. In 7 patients, sonographic signs of liver cirrhosis were detectable. Median TE values were 20.0 kPA [IQR 15.0, 34.3]. FALD was graded as mild in 6, moderate in 11 and severe in 22 patients based on the FALD score (7).

**Table 2:** Hepatic assessment.

	**Median [IQR]**	**Outside RR/N (%)**
**Laboratory parameters**		
ALT (U/l)	30.0 [25.0; 38.0]	6/39 (15.4%)
AST (U/l)	30.0 [26.0; 34.0]	7/39 (17.9%)
γGT (U/l)	87.0 [54.0; 125.0]	31/39 (79.5%)
Total bilirubin (mg/dl)	0.9 [0.7; 1.6]	13/39 (33.3%)
Thrombocytes (K/μl)	159.0 [137.0; 211.0.]	12/39 (30.8%)
**Fibrotest®**	0.6 [0.4; 0.7]	
F0		4/34 (11.8%)
F1		6/34 (17.6%)
F2		12/34 (35.3%)
F3		8/34 (23.5%)
F4		8/34 (23.5%)
**TE (kPa)**	20.0 [15.0; 34.3]	
<21.2 kPa		18/34 (52.9%)
21.3–27.6		2/34 (5.9%)
27.7–35.7		8/34 (23.5%)
> 35.8 kPa		7/34 (20.6%)
**Hepatic ultrasound findings**
Hepatomegaly		9/35 (25.7%)
Splenomegaly		9/35 (25.7%)
Heterogeneous liver parenchyma		35/35 (100.0%)
Segmental atrophy/hypertrophy		11/35 (31.4%)
Hepatic vein dilatation		31/35 (88.6%)
Abnormal hepatic vein architecture		35/35 (100.0%)
Hyperechogenic lesions		7/35 (20.0%)
Surface nodularity		7/35 (20.0%)
Ascites		4/35 (11.4%)
FALD score/ FALD severity	6.0 [5, 8]	
Mild		6/39 (15.4%)
Moderate		11/39 (28.2%)
Severe		22/39 (56.5%)
**Maximal liver function capacity**
LiMax® test (μg/kg*h)	350.0 [288.0; 470.0]	
Hepatic metabolic impairment		
None (> 315 μg/kg*h)		28/39 (71.8%)
Moderate (140–315 μg/kg*h)		11/39 (28.2%)
Severe (0–139 μg/kg*h)		0/39 (0.0%)

*Data are presented as median [IQR] or frequencies (%)*.

*AST, Aspartate aminotransferase; ALT, Alanine aminotransferase; γGT, γ-Glutamyl-aminotransferase; IQR, interquartile range; kPa, Kilopascal; RR, reference range; TE, Transient elastography*.

**Figure 1:**
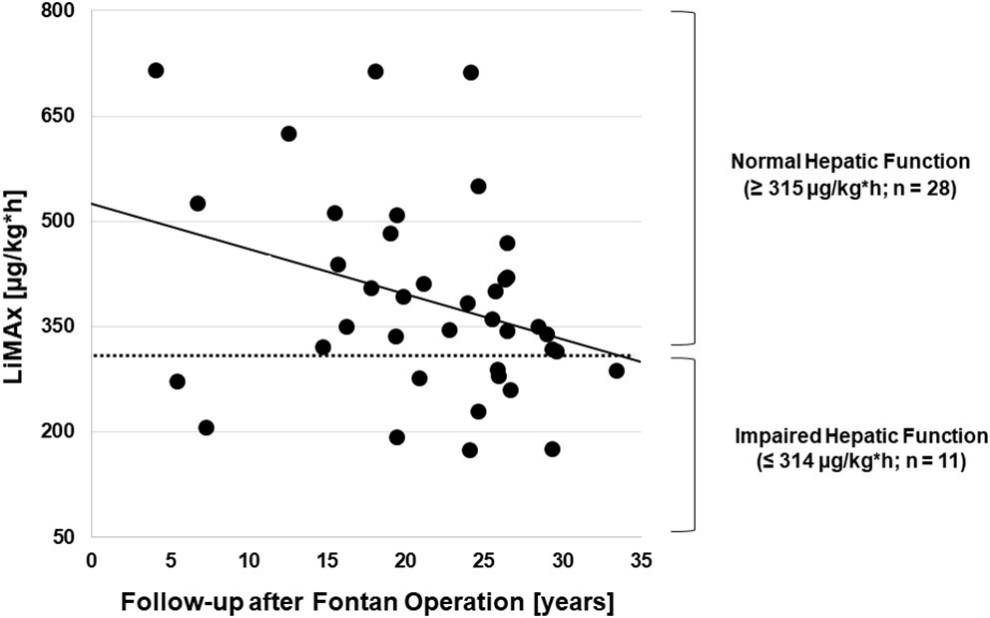
Correlation between the follow-up time after Fontan operation and maximal liver function capacity (*n* = 39).

Median maximal liver function capacity was 350.0 μg/kg^*^h [IQR 288.0; 470.0] corresponding to a normal hepatic function (≥ 315 μg/kg/h) based on previously published normative values (12). In 11 patients, a moderate hepatic impairment was detected (140–314 μg/kg^*^h), while none had severely impaired maximal liver function capacity (≤ 139 μg/kg^*^h). As shown in Figure 1, hepatic function as assessed by LiMax® tended to decrease with a longer follow-up after Fontan operation (*r*^2^ = −0.333, *p* = 0.038). A significant correlation between maximal liver function capacity and the laboratory parameters bilirubin (*r*^2^ = −0.421, *p* = 0.009) and γGT (*r*^2^ = −0.367; *p* = 0.021) was detected. However, no significant correlations were found between maximal liver function capacity and Fibrotest® fibrosis score (*r*^2^ = −0.335, *p* = 0.052), TE (*r*^2^ = −0.151, *p* = 0.388) or the number of hepatic abnormalities detected by liver ultrasound (*r*^2^ = −0.204, *p* = 0.24). Seven of thirty-nine patients were diagnosed with liver cirrhosis based on sonography, in these patients maximal liver function capacity was significantly reduced as compared to patients without liver cirrhosis (274.5 μg/kg^*^h [IQR 216.3; 346.0] vs. 384.0 μg/h^*^kg [IQR 320.0; 497.5], *p* = 0.025).

**Figure 2:**
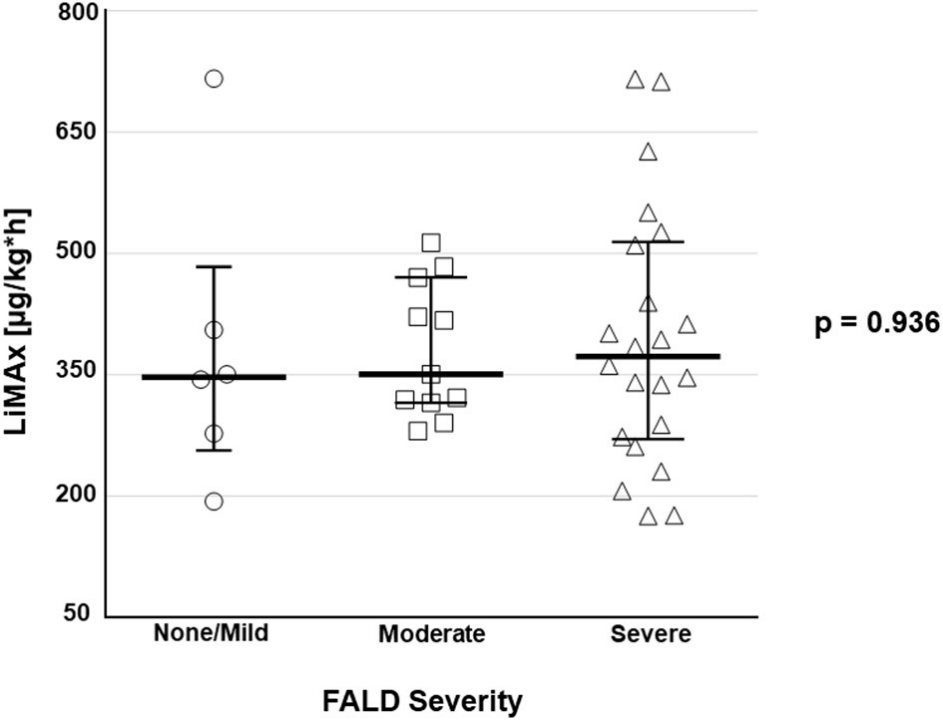
Boxplots depict maximal liver function capacity according to the classification of FALD severity [absent/ mild (*n* = 6), moderate (*n* = 11), severe (*n* = 22)].

No correlation was observed between maximal liver function capacity and the FALD score (*r*^2^ = −0.237; *p* = 0.152). Also, maximal liver function capacity did not differ significantly between varying degrees of FALD severity (*p* = 0.936; Figure 2).

## Discussion

Fontan-associated liver disease (FALD) encompasses all abnormalities in liver structure and function which are associated with the unphysiological Fontan circulation (3–6). Affecting up to 80% of the Fontan population, it is the most common second-organ disease (7–9). Various studies focused on the detection and monitoring of FALD, but its clinical significance and importance for therapeutic decision-making remains unclear. The LiMAx® test was successfully evaluated in several clinical settings such as hepatic surgery, hepatic transplantation, sepsis, and hepatic cirrhosis (10, 12–15). To the best of our knowledge, we herein provide the first results on maximal liver function capacity measured by LiMAx® in Fontan patients in comparison with other diagnostic modalities and FALD severity. Of note, maximal liver function capacity was normal in the majority of our patients although more than half of our cohort had FALD graded as severe. In addition, no clear correlation of maximal liver function capacity with TE and sonographic hepatic abnormalities was detected. We only found a relationship between the laboratory parameters bilirubin and γGT and LiMAx® values. An increase of laboratory parameters associated with cholestasis is the most common abnormality in Fontan patients as well as in patients with congestive hepatopathy (16). The degree of cholestasis is known to be related to increases in right atrial pressure and to the severity of tricuspid valve regurgitation in patients with right heart failure (17, 18). The underlying mechanism is supposed to be the compression of bile canaliculi and small ductules by centrally congested sinusoids (16). Additionally, diminished cardiac output results in reduced vascular supply and increased oxidative stress leading to necrosis of centrilobular hepatocytes and ischemic damage of the intrasinusoidal endothelium (16), which might not only be detected by increased indicators of cholestasis but also by reduced maximal liver function capacity.

The major finding of our study is the missing correlation between FALD severity and maximal liver capacity measured by the LiMAx® test. This important result suggests that the progress of hepatocyte-specific damage is decelerated and enzymatic liver function remains preserved for a considerably long time in the majority of patients during the morphologic development and progression of FALD. This finding may have important implications for the routine hepatic follow-up of Fontan patients and for the evaluation of failing Fontan patients for cardiac transplantation. Assessing maximal liver function capacity may help to identify failing Fontan patients that require a combined cardiac and liver transplantation. In line with these findings, we previously reported remarkable hepatic remodeling in a patient with normalization of hepatic stiffness values and regression of sonographic signs of hepatic cirrhosis after cardiac transplantation (7). This observation underlines that certain FALD-specific abnormalities are potentially reversible, which might be explained by the missing or slow progression of hepatic injury revealed by our measurements of maximal liver function capacity.

### Maximal Liver Function Capacity and Evaluation for Cardiac Transplantation

Cardiac transplantation is currently the only remaining treatment option for patients with refractory Fontan failure since effective evidence-based medical heart failure therapies are virtually non-existent, and mechanic circulatory support is not well-established in Fontan-palliated patients (19, 20). However, cardiac transplantation remains a high risk surgical procedure in the adult Fontan population and hepatic end-organ damage contributes to post-transplant morbidity and mortality (8, 9). Moreover, no guidelines exist for the optimal timing for cardiac transplantation in Fontan patients which may result in a delayed listing for transplantation with already advanced second-organ damage that may negatively impact transplantation outcome. In our cohort, maximal liver function capacity was significantly reduced in patients listed or evaluated for listing for cardiac transplantation (*n* = 4) compared to the remaining cohort (288.0 μg/kg^*^h [IQR 182.8, 331.5] vs. 361.0 μg/kg^*^h [IQR 302.5, 454.5], *p* = 0.035). This observation suggests that FALD was rather progressed in these patients, already resulting in functional impairment of the liver which in turn might indicate that listing for cardiac transplantation needs to be considered early. In a previous study, we emphasized on the importance of FALD monitoring to determine the optimal timing for cardiac transplantation and proposed the FALD score as a tool to grade FALD severity and facilitate surveillance of FALD progression (7). The LiMAx® test may represent a valuable complementary additional diagnostic modality in the hepatic assessment of Fontan patients since it provides a reproducible quantitative measurement of enzymatic hepatocyte function. Since a deterioration of maximal liver function capacity seems to occur relatively late during the disease course, its occurrence should trigger an evaluation for cardiac transplantation.

## Conclusion

We herein demonstrate that maximal liver function capacity as measured by the LiMAx® test is generally well-preserved in Fontan patients despite morphologic evidence of advanced FALD. Our findings suggest that the development and progression of FALD is not a uniform process and diagnostics of chronic hepatic injury during follow-up after Fontan operation should encompass various modalities. Specifically, morphologic changes as detected by ultrasound and/or TE seem to precede functional impairment as evidenced by preserved hepatic function in several patients with significant structural anomalies.

### Limitations

There are several limitations to this study. This is a cross-sectional single center trial with a comparably small patient cohort. Future studies, preferably in a multi-institutional setting, are necessary to comprehensively evaluate the assessment of hepatic function in Fontan palliated patients. Methacetin is only approved in adult patients, therefore pediatric patients could not be included in this study. After intravenous injection ^13^C methacetin undergoes a hepatic microsomal deacylation into paracetamol and ^13^CO_2_, which is transported to the lung as bicarbonate_._ Inter – and intraindividual bicarbonate kinetics might influence the respiratory ^13^CO_2_ excretion by delayed liberation of transiently trapped ^13^CO_2_. Moreover, although our results suggests an earlier appearance of morphologic changes as compared to functional impairments the temporal relationships of FALD progression and changes of maximal liver function capacity cannot be determined by our cross-sectional study design and requires longitudinal studies. Additional diagnostic modalities such as histological analyses from biopsies or hepatic magnetic resonance imaging were not included in our routine hepatic assessment and the relationship between Fontan hemodynamics and hepatic function was not addressed in this study and needs to be evaluated in the future. The FALD score and gradation of FALD severity have not been evaluated or validated in large patient cohorts or multicenter settings.

## Data Availability Statement

The raw data supporting the conclusions of this article will be made available by the authors, without undue reservation.

## Ethics Statement

The studies involving human participants were reviewed and approved by Ethikkommission der Charité-Universitätsmedizin Berlin (desicion number EA2/127/18). The patients/participants provided their written informed consent to participate in this study.

## Author Contributions

AS and SO: conceptualization. AS, NJ, and MP: data collection. AS, FD, PK, MS, HS, H-PM, and TM: investigation. AS: formal analysis and writing original draft. SO, FB, FT, MJ, and MS: supervision. SO, PK, FB, FT, HS, and TM: writing review and editing. All authors contributed to the article and approved the submitted version.

## Conflict of Interest

The authors declare that the research was conducted in the absence of any commercial or financial relationships that could be construed as a potential conflict of interest.

## Publisher's Note

All claims expressed in this article are solely those of the authors and do not necessarily represent those of their affiliated organizations, or those of the publisher, the editors and the reviewers. Any product that may be evaluated in this article, or claim that may be made by its manufacturer, is not guaranteed or endorsed by the publisher.

## References

[1] d'Udekem Y Iyengar AJ Galati JC Forsdick V Weintraub RG Wheaton GR. Redefining expectations of long-term survival after the Fontan procedure. Twenty-five years of follow-up from the entire population of Australia and New Zealand. Circulation. (2014) 130:32–8. 10.1161/CIRCULATIONAHA.113.007764 25200053

[2] Pundi KN Johnson JN Dearani JA Pundi KN Li Z Hinck CA. 40-year follow up after the Fontan operation. Long-term outcomes of 1052 patients. J Am Coll Cardiol. (2015) 66:1700–10. 10.1016/j.jacc.2015.07.065 26449141

[3] Rychik J Veldtman G Rand E Russo P Rome JJ Krok K. The precarious state of the liver after a Fontan operation: summary of a multidisciplinary symposium. Pediatr Cardiol. (2012) 33:1001–12. 10.1007/s00246-012-0315-7 22534759 PMC3442163

[4] Goldberg DJ Surrey LF Glatz AC Dodds K O'Byrne ML Lin HC. Hepatic fibrosis is universal following Fontan operation, and severity is associated with time from surgery: a liver biopsy and hemodynamic study. J Am Heart Assoc. (2017) 6:e004809. 10.1161/JAHA.116.004809 28446492 PMC5524062

[5] Schleiger A Salzmann M Kramer P Danne F Schubert S Bassir C. Severity of Fontan-associated liver disease correlates with Fontan hemodynamics. Pediatr Cardiol. (2020) 41:736–46. 10.1007/s00246-020-02291-5 32006084 PMC7256101

[6] Musterman ID Duijnhouwer AL Kendall TJ Bronkhorst CM Ronot M vanWettere M. The clinical spectrum of Fontan-associated liver disease: results from a prospective multimodality screening cohort. Eur Heart J. (2019) 40:1057–68. 10.1093/eurheartj/ehy620 30346512

[7] Schleiger A Kramer P Salzmann M Danne F Schubert S Bassir C. Evaluation of Fontan failure by classifying the severity of Fontan-associated liver disease - a single centre cross-sectional study. Eur J Cardiothorac Surg. (2020) 59:341–8. 10.1093/ejcts/ezaa310 33111145

[8] Berg CJ Bauer BS Hageman A Aboulhosn JA Reardon LC. Mortality risk stratification in Fontan patients who underwent heart transplantation. Am J Cardiol. (2017) 119:1675–9. 10.1016/j.amjcard.2017.02.005 28341356

[9] Polyviou S O'Sullivan J Hasan A Coats L. Mortality risk stratification in small patients cohorts: The post-Fontan heart transplantation paradigm. Am J Cardiol. (2018) 122:182–8. 10.1016/j.amjcard.2018.03.021 29759294

[10] Stockmann M Lock JF Riecke B Heyne K Martus P Fricke M. Prediction of postoperative outcome after hepatectomy with a new bedside test for maximal liver function capacity. Ann Surg. (2009) 250:119–25. 10.1097/SLA.0b013e3181ad85b5 19561474

[11] De Lédinghen V Le Bail B Rebouissoux L Fournier C Foucher J Miette V. Liver stiffness measurement in children using FibroScan: Feasibility study and comparison with Fibrotest, aspartate transaminase to platelets ratio index, liver biopsy. JPGN. (2007) 45:443–50. 10.1097/MPG.0b013e31812e56ff 18030211

[12] Stockmann M Lock JF Malinowski M Niehues SN Seehofer D Neuhaus P. The LiMAx test: a new liver function test for predicting postoperative outcome in liver surgery. Hpb. (2010) 12:139–46. 10.1111/j.1477-2574.2009.00151.x 20495659 PMC2826673

[13] Jara M Malinowski M Lüttgert K Schott E Neuhaus P Stockmann M. Prognostic value of enzymatic liver function for the estimation of short-term survival of liver transplant candidates: a prospective study with the LiMAx test. Transplant Int. (2015) 28:52–8. 10.1111/tri.12441 25263095

[14] Kaffarnik M Lock JF Vetter H Ahmadi N Lojewski C Malinowski M. Early diagnosis of sepsis-related hepatic dysfunction and its prognostic impact on survival: a prospective study with the LiMax test. Crit Care. (2013) 17:R259. 10.1186/cc13089 24172237 PMC4057158

[15] Malinowski M Jara M Lüttgert K Orr J Lock JF Schott E. Enzymatic liver function correlates with disease severity of patients with liver cirrhosis: a study with the LiMAx test. Dig Dis Sci. (2014) 12:2983–91. 10.1007/s10620-014-3250-z 24993690

[16] De Gonzalez AKK Lefkowitch JH. Heart disease and the liver: pathologic evaluation. Gastroenterol Clin N Am. (2017) 46:421–35. 10.1016/j.gtc.2017.01.012 28506373

[17] Megalla S Holtzman D Aronow WS Nazari R Korenfeld S Schwarcz A. Predictors of cardiac hepatopathy in patients with right heart failure. Med Sci Monit. (2011) 17:CR537-41. 10.12659/MSM.881977 21959605 PMC3539469

[18] Lau GT Tan HC Kritharides L. Type of liver dysfunction in heart failure and its relation to the severity of tricuspid regurgitation. Am J Cardiol. (2002) 90:1405–9. 10.1016/S0002-9149(02)02886-2 12480058

[19] Ghanayem NS Berger S Tweddell JS. Medical management of the Failing Fontan. Pediatr Cardiol. (2007) 28:465–71. 10.1007/s00246-007-9007-0 17763892

[20] Miller JR Lancaster TS Callahan C Abarbanell AM Eghtesadi P. An overview of mechanical circulatory support in single-ventricle patients. Transl Pediatr. (2018) 7:151–61. 10.21037/tp.2018.03.03 29770296 PMC5938256

