# Supplementary material for: Morphologic Alterations Precede Functional Hepatic Impairment as Determined by 13C-Methacetin Liver Function Breath Test in Adult Fontan Patients
Source: Front Cardiovasc Med. 2021 Dec 23;8:764009. doi: 10.3389/fcvm.2021.764009 (PMC8732997; doi:10.3389/fcvm.2021.764009)
Supplement: Supplementary file 1 [file Data_Sheet_1.docx]

Supplemental Material

**
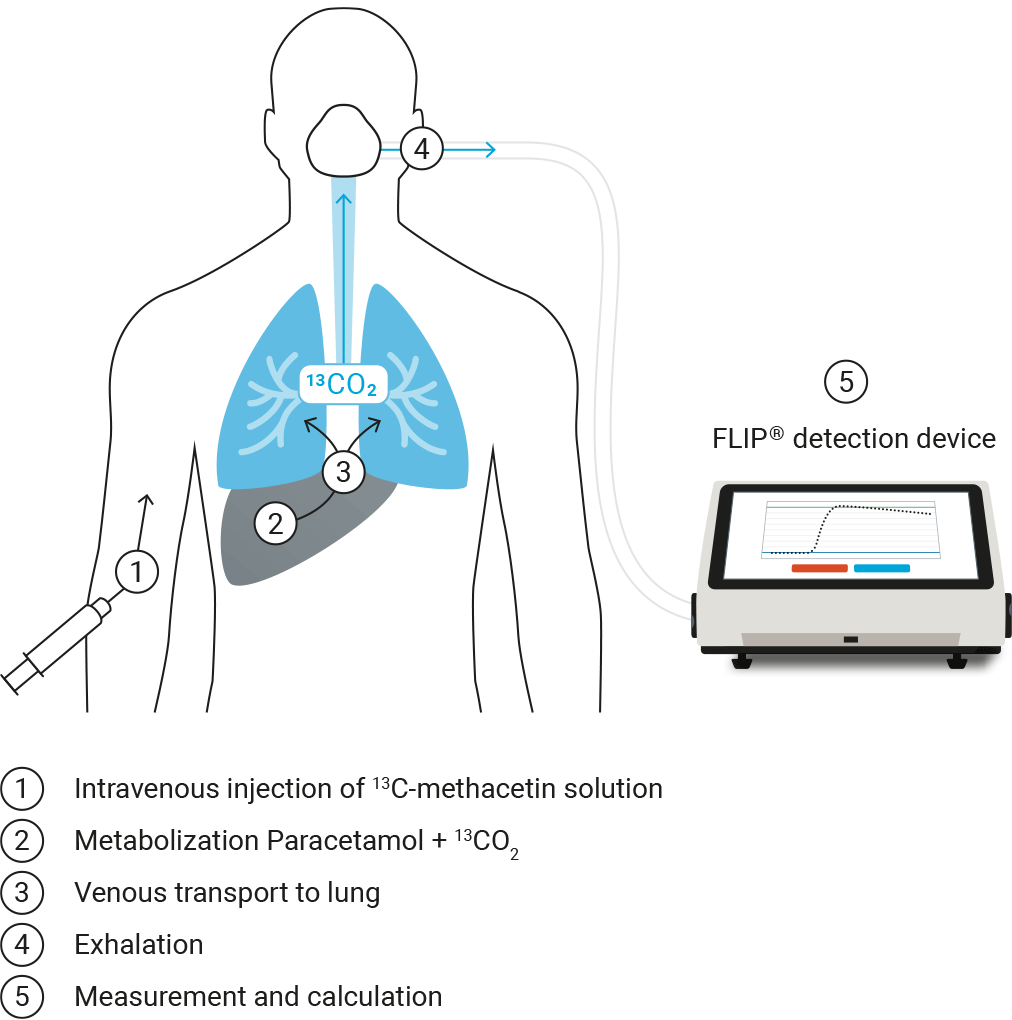
**

**Supplemental Figure 1.** Schematic diagram for the performance of the LiMAx® test. <https://www.humedics.eu/en/why-limax.html>.

A


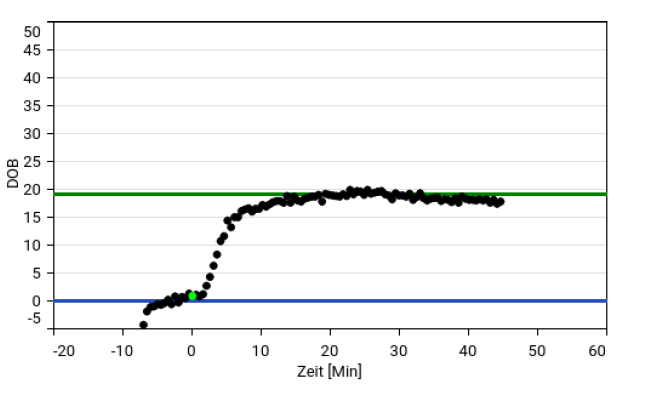


B

time [min]

time [min]

**Supplemental Figure 2.** Example of the result of maximal liver function capacity measured by the LiMAx® test in a Fontan patient with normal (A) and moderately impaired hepatic function (B). The blue line represents the base line and the green line the median of delta-over-baseline (DOB) calculation of the measured ^13^CO_2_/^12^CO_2_ ratio.
